# The Importance of the PI3K/AKT/MTOR Pathway in the Progression of Ovarian Cancer

**DOI:** 10.3390/ijms14048213

**Published:** 2013-04-15

**Authors:** Zachary C. Dobbin, Charles N. Landen

**Affiliations:** Division of Gynecologic Oncology, Department of Obstetrics and Gynecology, University of Alabama at Birmingham, Wallace Tumor Institute, 1824 6th Avenue South, Birmingham, AL 35233, USA; E-Mail: zdobbin@uab.edu

**Keywords:** ovarian cancer, PI3K, mTOR, AKT

## Abstract

Ovarian cancer is the fifth most common cause of death due to cancer in women despite being the tenth in incidence. Unfortunately, the five-year survival rate is only 45%, which has not improved much in the past 30 years. Even though the majority of women have successful initial therapy, the low rate of survival is due to the eventual recurrence and succumbing to their disease. With the recent release of the Cancer Genome Atlas for ovarian cancer, it was shown that the PI3K/AKT/mTOR pathway was one of the most frequently mutated or altered pathways in patients’ tumors. Researching how the PI3K/AKT/mTOR pathway affects the progression and tumorigensis of ovarian cancer will hopefully lead to new therapies that will increase survival for women. This review focuses on recent research on the PI3K/AKT/mTOR pathway and its role in the progression and tumorigensis of ovarian cancer.

## 1. Introduction

Ovarian cancer is the fifth most common cause of death due to cancer in women, despite ranking tenth in incidence [1]. In ovarian cancer, primary treatment is surgical resection of visible disease followed by adjuvant chemotherapy usually consisting of a combination of platinum-based and taxane-based chemotherapy. Currently, the five-year survival rate for ovarian cancer is only 45% [1]. This high mortality rate is due to the high incidence of patients presenting with advanced stage disease and the high rate of recurrence despite successful initial therapy. Approximately 50% of all patients treated with first-line chemotherapy will achieve a complete clinical response; however, if those patients undergo a secondary laparotomy, 50% of the complete clinical response patients will be positive for residual disease, and rarely ever be disease-free [2]. Importantly, even among patients who have no visible or pathologically detected disease, 50% of those will eventually relapse. This leads to the somber fact that more than 70% of patients will ultimately develop recurrent disease [2].

In order to reduce the high mortality rate seen in ovarian cancer, research is being conducted in early detection [3–5] and in development of new therapeutics to treat recurrence and chemoresistance in ovarian cancer. In terms of treatment of ovarian cancer, many clinical trials have focused on changing the dosing, scheduling, and combination of available chemotherapies in order to improve survival. While there have been moderate improvements, such as using intraperitoneal delivery of chemotherapy, or dose-dense Taxol regimens, cure rates have not changed significantly. Therefore, in order to improve survival, new therapeutics need to be developed that will target the chemoresistant population of ovarian cancers.

In ovarian cancer, numerous targeted therapies have been developed and tested with limited success. This indicates that identification of an advanced ovarian cancer depending on a single gene or on oncogene addiction that can be targeted by a single agent is rare [6]. Furthermore, there is prevailing evidence that ovarian cancers can be broadly classified into two groups, type I and type II. Type I ovarian cancer is considered low grade and will more often present in an early stage but still have relative resistance to platinum-based therapy. Type II ovarian cancers are represented by high grade serous and undifferentiated cancers that present at a late stage and, while aggressive, normally initially respond to platinum-based therapy [6].

The Cancer Genome Atlas has identified numerous activating mutations, DNA copy number changes and inactivating mutations in ovarian cancer that demonstrate the complex heterogeneity seen in ovarian cancer. While this complexity indicates that there will likely never be one molecular-targeted therapy that will cure all ovarian cancer, several pathways are frequently abnormal. One such pathway is the PI3K/AKT/mTOR pathway, with mutations or amplifications in 34% of samples analyzed [7]. These include mutations in *PIK3CA*, deletion in *PTEN*, amplification of *AKT1*, *AKT2*, and *AKT3*, which all lead to an aberrant functioning PI3K/AKT/mTOR pathway. In this review, the focus will be on recent research implicating the PI3K/AKT/mTOR pathway in ovarian cancer progression and tumorigenesis.

## 2. Overview of the PI3K/AKT/mTOR Pathway

The PI3K/Akt/mTOR pathway is a central regulator in both normal cell physiology and in cancer proliferation, tumorigenesis, and metastasis. The pathway is comprised of three main driving molecules: PI3 kinase (PI3K), AKT, and mammalian target of rapamycin (mTOR).

The PI3K are a family of lipid kinases that phosphorylate the 3-hydroxyl group of phosphoionositides [8]. There are three classes that make up the PI3K family: class I, class II, and class III [9]. Class I are heterodimers of PI3K consisting of a catalytic p110 subunit and a regulatory p85 subunit. The p110 has three isoforms (α, β, and δ). A combination of the p85 subunit and the p110 (α, β or δ) make up the group known as class IA PI3K. Class IB is made up of a p101 and 110-γ subunit [8]. Together, the role of class 1 PI3K is involved in cell proliferation, insulin signaling, immune function and inflammation [8,9]. Class II PI3Ks are monomeric catalytic isoforms involved in the regulation of membrane trafficking, while class III, solely made up of Vps34, has a role in autophagy [10]. It is primarily class IA PI3K that has been implicated in cancer and have numerous targeted pharmaceuticals being developed or currently in clinical trials.

After PI3K is fully activated, the kinase converts the substrate phosphatidylinositol 4,5-bisphosphate (PI(4,5)-P2) into PIP(3,4,5)3. This conversion of PIP2 to PIP3 allows for AKT and PDK1 to be brought together near the inside of the cell membrane. This results in AKT, a serine/threonine kinase, being phosphorylated at threonine-308 in its kinase domain. AKT can also be activated by phosphorylation at serine-473 by mTOR-Rictor (MTORC2) which is in the helical domain of AKT [11]. AKT is the central molecule in the PI3K/AKT/mTOR pathway, activating and modulating numerous downstream targets. AKT can stimulate protein synthesis and cell growth by activating mTOR though inhibition of the TSC1/2 complex and modulating cell proliferation by inactivating cell cycle inhibitors [9,12,13].

TOR was originally discovered in the yeast *Saccharomyces cervisiae* by the observation that this protein was inhibited by the macrolide rapamycin [14]. Later, a structurally and functional conserved mammalian version was discovered and designated as mTOR [15,16]. mTOR is a 289 kDa serine/threonine kinase that actually belongs to the PI3K-related protein kinase family as its *C*-terminus shares strong homology to the catalytic domain of PI3K [16]. In the mammalian cell, it was discovered that mTOR actually exists in two complexes, mTORC1 and mTORC2 [17,18]. MTORC1 is made up of raptor, mTOR, PRAS40, mLST8/GβL, and deptor, while mTORC2 contains rictor, mTOR, mLST8/GβL, Sin1, protor-1, and deptor [17,19]. MTORC2 is unique from MTORC1 not only because of the slight difference in molecules that make up the complex but because it is not sensitive to rapamycin [17]. mTORC1 is sensitive to growth factor stimulation, oxygen levels, or nutrient availability and functions by regulating the phosphorylation of rS6K and 4E-BP1, two proteins involved in the control of protein synthesis, translation initiation, and cell mass. mTORC2 participates in cell survival and proliferation in part through its ability to control AKT activity by phosphorylation of AKT at serine-473 [11].

The role of the PI3K/Akt/mTOR pathway in ovarian cancer is foreshadowed by its role in protecting the primordial follicles from destruction during normal oocyte maturation. Polycyclic aromatic hydrocarbons, which are environmental toxins, are known reproductive toxins that results in primordial follicle atresia causing premature ovarian failure [20]. One polycyclic aromatic hydrocarbon that has been shown to induce ovotoxicity is 3-methylcholanthrene (3MC) [20,21]. However, until recently, the mechanism was not well understood. When murine ovaries are treated with 3MC, it has been documented that follicular atresia [22] can be prevented with treatment of LY294002, a PI3K inhibitor. In the face of insult by an ovotoxin, the follicles attempt cell survival via upregulation of the PI3K/AKT/mTOR pathway that paradoxically leads to an increase in follicular proliferation, depleting the reserve of primordial ovarian follicles [22]. This results in the phenotype of premature ovarian failure in 3MC treatment. As in cancer, the PI3K/Akt/mTOR pathway has a key role in promoting cell survival in the normal ovary.

The role of the PI3K/AKT/mTOR pathway in ovarian cancer is extremely complex, arising from two main sources: (1) the diverse alterations found with PI3K/AKT/mTOR pathway itself; and (2) the diverse alterations in inputs into the PI3K/AKT/mTOR pathway. Through these various changes, the PI3K/AKT/mTOR pathway has demonstrated to play a key role in ovarian cancer tumorigenesis, progression, and chemotherapy resistance.

## 3. Tumorigenesis of Ovarian Cancer and PI3K/AKT/mTOR Pathway

Historically, the subtypes of epithelial ovarian cancer have been defined by histology and are primarily classified into papillary serous, endometrioid, mucinous, and clear cell [23,24]. Recent evidence is leading to the idea that the disease of epithelial ovarian cancer is actually comprised of a spectrum of cancer types that originate from different pelvic organs, most notably form the fallopian tube [6,25,26].

While Kim *et al.* identified a role for PI3K/AKT/mTOR in the tumorigenesis of type II ovarian cancer arising from the fallopian tube, other groups have implicated the pathway in the tumorigenesis of type I ovarian cancer arising from the ovarian bursa. Type I ovarian cancer is considered lower grade than type II and typically less responsive to traditional chemotherapy [27]. In addition, Type I has frequent cell signaling pathway mutations in KRAS, BRAF, CTNNB1, and PTEN and comprises most endometrioid, clear cell, and mucinous histologies [28,29]. When *Apc* and *Pten* are conditionally inactivated in the ovarian bursa of a mouse, an endometrioid ovarian carcinoma develops that has nuclear expression of β-catenin and absence of PTEN expression [27].

While the above models required one mutation in PI3K/Akt/mTOR coupled with a mutation in another pathway, if a double knockout is present with alterations to two members of the PI3K/AKT/mTOR pathway, ovarian tumorigenesis can occur. Using a genetically engineered mouse that was bred to have an activating *PIK3CA^H1047R^* mutation and be *Pten^WT/del^*, Kinross *et al.* noticed that the mice only had hyperplasia of the ovarian surface epithelium [30]. However, when a second deletion of *Pten* was introduced directly into the ovarian bursa, the mice developed ovarian serous adenocarcinomas and granulosa cell tumors. This indicates that a secondary defect in a co-regulator of PI3K activity is sufficient in conjunction with a mutant *PIK3CA* for tumorigenesis to occur [30]. Mutations in the PI3K/AKT/mTOR pathway clearly result in the generation of ovarian tumors; however, what type they relate to clinically depends on the type of the genetic loss and the combination of genetic mutations.

## 4. PI3K/AKT/mTOR in the Proliferation and Progression of Ovarian Cancer

The role of the PI3K/AKT/mTOR pathway in terms of proliferation and progression of ovarian cancer is extremely complex. Many perturbations have been shown to contribute to carcinogenesis, with the endpoint the same: activation of the pathway results in an increase in cell proliferation, migration, invasion, and chemotherapy resistance.

The complexity begins with how deregulation of PI3K/AKT/mTOR can occur as a result of over-activation, mutations in the catalytic domains, mutations in the regulatory domain, or modifications to the downstream targets of PI3K. As demonstrated by the TCGA, the most prevalent mutational alterations are those affecting *PIK3CA* and *PTEN*[7]. *PTEN* is located on chromosome 10q23 and functional loss of *PTEN* impairs its lipid phosphatase activity, which is critical for tumor suppressor activity [31]. For *PIK3CA*, its dysfunction arises as a mutation on chromosome 3 that is predominately observed in endometrial, breast, and colorectal cancers or by gene amplification in ovarian cancer [32].

Robust pre-clinical models have been established for studying the PI3K/AKT/mTOR pathway in ovarian cancer. For example, SKOV3 has an activating mutation in *PIK3CA*[33] and the A2780 cell line has deletion of *PTEN*[34]. By targeting the individual members of the PI3K/AKT/mTOR pathway with siRNA, the role of each component can be easily elucidated.

If the p100 subunit of PI3K, which is encoded for by *PIK3CA*, is targeted with siRNA in OVCAR-3 cells, there is a decrease in migration, decreased invasion, and a decrease in proliferation [35]. The decrease in proliferation has also been replicated in OVCAR-8 (*AKT2* copy number gain), UPN251 (*PIK3CA* DNA copy number gain) and A2008 (*PIK3CA* mutation) cell lines that are treated with siRNA against PIK3CA [36]. However, one report was not able to reduce proliferation in OVCAR-3 cells treated with the PI3K inhibitor LY29400 [37]. The difference might be accounted for given molecular-targeted therapies require the over-activation of the target in order for the therapy to have a target. While OVCAR-3 may have low basal AKT activity, targeting it via siRNA will still knockout any expression [35,37]. This leads to the complexity in designing treatments that take advantage of the pathway in ovarian cancer. Overall targeting of *PIK3CA* results in the decrease of proliferation markers CyclinD1, CDK4, CyclinE, CDK2 and p21 and an increase in expression of p27. As G1 cell cycle progression is regulated by the CDK inhibitor p27, the release from its inhibition seems to account for the decrease in cell proliferation [35].

Proliferation and invasion is also affected when AKT is directly targeted as well. SiRNA against the AKT1 isoform reduces proliferation of OVCAR-3 cells, but to a lesser degree than inhibition of PIK3CA [35]. Targeting the AKT2 isoform has been shown to increase the activation of apoptosis [36]. This increase in apoptosis activation is not seen when PIK3CA is targeted. Invasion of ovarian cancer cells is reduced with AKT1 knockout but to a lesser extent then PIK3CA knockout [35,36]. When p110-α or AKT1are targeted with siRNA, there is also a decrease in the downstream molecule p70S6K1. Directly targeting p70S6K1 also reduces proliferation and invasion in ovarian cancer cells, though there is no rescue of expression of the CDK-inhibitor p27^KIP1^ that is seen in targeting p100-α or AKT1 [35]. This indicates the cell cycle is not being inhibited as strongly as when molecules higher in the PI3K/AKT/mTOR pathway are targeted.

Targeting mTOR directly can also decrease ovarian cancer cell proliferation and migration. However, the complexity of mTOR in the pathway contributes to the difficulty in elucidating mTOR’s exact role in proliferation. As mentioned earlier, mTOR can be found in two complexes: MTORC1 and MTORC2 [17–19]. It is important to study each complex independently as treating with rapamycin shows a differential response in each complex. When mTORC1 was targeted using siRNA against raptor, there was a decrease in pS6 and p4E-BP1 levels [17]. Raptor knockdown also provokes an increase in pS^473^-AKT, indicating compensatory activation of AKT by mTORC2 in response to loss of mTORC1 signaling. Conversely, rictor knockdown decreases pS^473^-AKT and pS6 levels. In terms of proliferation, knockdown of raptor has a greater inhibitory effect then knockdown of rictor. Raptor has a similar effect on proliferation as mTOR siRNA knockdown, thereby indicating that mTORC1 is more important in cell proliferation for ovarian cancer [17]. Though MTORC1 signaling has the more important role in ovarian cancer cell proliferation than MTORC2, therapeutically, both molecules will need to be targeted to prevent the compensatory activation of AKT via MTORC2 when MTORC1 is inhibited alone [17,38].

While the activation of PI3K/AKT/mTOR leads to an increase in proliferation, invasion, and migration, the mechanism of how this occurs appears to be regulated through essential matrix metalloproteinase (MMPs). MMPs are zinc-dependent endopeptidases with the ability to degrade various extracellular matrix proteins. They are involved in cleavage of cell surface receptors and releasing apoptotic signals and by targeting collagen IV in the basement help allow a cell to migrate [39,40]. Tissue inhibitor of matrix metalloproteinases (TIMP) are naturally occurring inhibitors of MMPs, except for TIMP1 and TIMP2, which help activate MMP-2 and MMP-9 [41], thereby playing a role in migration and invasion in ovarian cancer [42]. Research in other malignancies has identified that activation of PI3K leads to an increase in MMP-2 activity and an increase in cell motility [43,44]. Treating ovarian cancer cell lines SKOV3, OVCAR5 and IGROV1 with a PI3K inhibitor, LY294002, there is a reduction in gondatropin-induced MMP-2 activity with little change in MMP-9 activity [42]. However, migration and invasion are significantly decreased when cells are treated with LY294002 and cisplatin. This can be occurring due to TIMP1 and TIMP2 expression decreased by LY294002 hence preventing migration through a decrease in MMP-2 activity [42].

Other studies have found that MMP-9 activity and not MMP-2 activity is responsible for migration and invasion. The flavonoid apigenin, which can inhibit tumor growth [45], is able to reduce the amount of metastases in the abdominal organs of an orthotopic xenograft model [46]. Mechanistically, the reduction in metastases is due to apigenin inhibiting AKT phosphorylation and subsequently causing a decrease in MMP-9 activity, though not MMP-2. While these results are in opposition to what Karam *et al.*[42] found—they saw a decrease in MMP-2 activity in the presence of a decrease in AKT phosphorylation—overall, the observed phenotype is identical. Whether through a decrease in MMP-2 or MMP-9 activity, inhibiting AKT phosphorylation results in a decrease in invasion, migration, and metastasis of ovarian cancer cells.

When brought together, a picture begins to emerge on how the PI3K/AKT/mTOR pathway is playing a key role in invasion for ovarian cancer. PI3K activation leads to the phosphorylation of AKT, which in turn activates p70S6K1. This downstream activation results in TIMP1 and TIMP2 expression activating MMP-2 or MMP-9 allowing for invasion and migration.

## 5. Outside Influences on the PI3K/AKT/mTOR Pathway

Numerous different inputs into the PI3K/AKT/mTOR pathway add to the complexity of the picture. One input involves the normal stress response pathway. The AMP-activated protein kinase (AMPK) is a metabolic stress-related and energy censor kinase that plays a role in monitoring the AMP/ATP ratio. Activation of AMPK ultimately results in downstream signals that control processes important for regulation of metabolism including fatty acid oxidation and mRNA translation/protein synthesis [47]. AMPK can suppress the activation of the mTOR pathway via indirect inhibitory effects on the mTORC1 complex by the phosphorylation and activation of the TSC2-TSC1 complex [47]. Normally AMPK physiologically inhibits mTOR in the context of decreased energy sources to the cell. However in cancer, there is evidence that AMPK signaling is reduced, allowing the cancer cell to escape normal proliferation controls [47]. While AMPK signaling is reduced in ovarian cancer cells, it can be restored though the use of metformin. Metformin is used in therapy for diabetes and can modulate AMPK activation [48]. With metformin treatment, AMPK activation inhibits protein biosynthesis and decreases phosphorylation of mTOR [48]. This results in a modulation of p21, p27, and Cyclin D1, thus reducing proliferation [48].

Alternatively, PI3K/AKT/mTOR can be activated by the loss of sMEK1 without affecting individual members of the PI3K/AKT/mTOR pathway. sMEK1 is a tumor suppressor of the protein phosphatase 4 regulatory subunit 3 (PP4R3) and the PP2A subfamily, which are conserved serine/threonine phosphatase [49]. Interestingly, sMEK1 is downregulated in ovarian and cervical tumor tissue [49]. However, re-expression of sMEK1 in the OVCAR-3 cell line results in a suppression of cell proliferation by inducing cell cycle arrest at G_1_/G_0_ phase with an increase in CDK inhibitor p16, and p27 [49]. In addition, sMEK1 expression induces PI3K and AKT dephosphorylation and reduction of expression of the mTOR/p70S6K proteins [49].

The PI3K/AKT/mTOR pathway can also be activated via alternative phosphorylation of AKT. PI3K will activate AKT through phosphorylation on a Threonine-308 residue. However, EGFR can phosphorylate AKT independent of PI3K change at the Serine-473 residue resulting in AKT over-activation [50] and an increase in angiogenesis, metastasis and anti-apoptosis properties. Reduction of EGFR phosphorylation and AKT phosphorylation results in apoptosis induction and the dissociation of rictor and raptor from mTOR, causing a decrease in proliferation [50]. The activation of the EGFR-AKT axis may also be further upstream involving G protein-coupled receptor 30 (GPR30). GPR30, a 7-transmembrane estrogen receptor, is widely expressed in cancer cell lines [51,52] and strongly associated with proliferation, invasion, metastasis, and drug resistance of various cancer cell lines [53–55]. GPR30 can phosphorylate EGFR and thus activate AKT in ovarian cancer cell [56], and at least one study has demonstrated a poor prognosis with high GPR30 expression [57].

Another example of mutations outside the pathway impacting mTOR is hyper-activation of fatty acid synthase (FASN). FASN is an enzyme responsible for *de novo* synthesis of lipids from sugars, is overexpressed in 80% of ovarian carcinomas [58], and has been shown to be a predictor of poor survival [59]. Inhibition of FASN results in PI3K and downstream mediators to be targeted for degradation by ubiquitilation, leading to cytoreduction and growth arrest in A2780, SKOV3, OVCAR-3 ovarian cancer cell lines [60]. This is unique, as pathway inhibitors usually result in a reduction of phosphorylation and not an actual decrease in measureable protein.

These outside pathways that result in activation of the PI3K/AKT/mTOR pathway will make it difficult to identify which patients would benefit the most from target therapy. If only the mutation status of PI3K/AKT/mTOR members is analyzed, potential candidates for treatment would be missed if their pathway aberration is the result of an outside influence.

## 6. MicroRNA, Ovarian Cancer, and the PI3K/AKT/mTOR Pathway

The discovery of miRNAs in 1993 and later their functional roles in cancer as tumor suppressors or oncogenes opened up a new understanding of tumorigenesis and possible therapeutic options [61,62]. Work by Zhang *et al*. in 2008 [63] brought some of the first research implicating miRNAs in the pathogenesis and tumorigenicity of epithelial ovarian cancer. miRNAs in ovarian cancer play both oncogenic and tumor suppressive roles. miRNA-93 has been identified as a regulator of PTEN/AKT signaling with expression of miRNA-93 inversely correlating with PTEN expression [64]. In addition, miRNA-93 has been shown to decrease cisplatin chemosensitivity in ovarian cancer cells. miRNA-21 also acts on ovarian cancer via the PI3K/AKT/mTOR pathway by suppressing PTEN, and is even activated by AKT in hypoxic conditions to induce survival [65,66]. Another oncogenic miRNA in ovarian cancer is miRNA-182, which has been shown to promote cell growth, invasion and chemoresistance by targeting programmed cell death 4 (PDCD4) and was able to reduce chemosensitivity of ovarian cancer cells to Taxol [67].

The role of miRNA in epithelial ovarian cancer is not always oncogenic. miRNA-152 and miRNA-185 co-contribute to cisplatin sensitivity in ovarian cancer cells [68]. Overexpressing miRNA-152 and miRNA-185 in cisplatin-resistant ovarian cancer cell lines results in restoring of chemosensitivity through suppressing DNA methytransferase 1 [68]. Research has indicated that miR-204 has an important role in tumorigenesis [69,70], and high-resolution custom miRNA comparative genomic hybridization has shown that there is frequent genomic loss in the chromosome containing miR-204 [71]. miR-204 is lost in 44.63% of ovarian tumors analyzed and its overexpression in SKOV3 ovarian cancer cells reduces colony-forming capacity. It appears that miR-204 is targeting genes associated with tumorigenesis [71] by reducing p-AKT, p-4E-BP1 and p-S6. However, the regulation of p-AKT is outside of the known phosphorylated residues associated with the PI3K/AKT/mTOR pathway [71]. This indicates miR-204 is downregulating p-AKT outside of the two phosphorylated residues known in to be activated in the PI3K/AKT/mTOR pathway.

## 7. Clinical Relevance of PI3K/AKT/mTOR Pathway

While preclinical studies have contributed invaluable knowledge about the progression and tumorigenesis of ovarian cancer, the final step is finding a correlation in the clinic. Not only are genetic alterations identified and observed in preclinical models present in clinical samples, but there is also prognostic and potential therapeutic value in understanding how a patient’s tumor has a modified PI3K/AKT/mTOR pathway. Analyses of clinical samples have ranged from looking at the mutational status of the regulator molecules of the pathway, changes in the activity of the downstream molecules, and the effect these changes have on survival and therapy options.

One molecule that is frequently mutated in ovarian cancer is *PIK3CA*. A mutational change here can result in over-activation of PI3K kinase activity. When there is a *PIK3CA^H1047R^* mutation, which is in the kinase domain, it results in enhanced lipid kinase activity [30]. If an inactivating mutation occurs in *PIK3R1*, the p85 regulatory subunit of PI3K, PI3K/AKT/mTOR over-activation occurs. In patients with a *PIK3CA* activating mutation, 40% also had an inactivating mutation in the regulatory genes *PIK3R1* or *PTEN*[30]. Mutations and alterations also occur in *AKT* resulting in an increased amount of activated AKT. Typically, ovarian cancer will have *AKT* amplification and at a lower frequency due to a missense mutation in *AKT*[35,72].

In a more comprehensive analysis of 93 primary ovarian tumors, Comparative Genomic Hybridization (aCGH) was used to identify copy number changes. When looking at nine canonical signaling pathways (PI3K/AKT/mTOR, MAPK, TGF-B, p38/MAPK, JNK, JAK/STAT, WNT/β-Catenin, and NFκB) and copy number variation in terms of patient survival, the PI3K/AKT/mTOR pathway was the most frequently altered cancer related pathway [36]. Similar to the findings of Kinross *et al.*, 40% of patients had genetic aberrations in *PIK3CA*, with the most copy number gains seen in all the patient samples [36]. The second most copy number gains were seen in *PIK3CB* (27%) and *Cyclin-D2* (27%), which would account for uncontrolled cell cycle progression in ovarian cancer [36]. Also *PIK3R4* and *PIK3R1*, genes for the regulatory subunit for PI3K, showed a decrease in copy number in 20% and 22% of patients, respectively [36]. Importantly, the copy number variations identified in the PI3K/AKT/mTOR pathway were directionally concordant with the expected oncogenic activity. This was not the case in the other pathways examined. In the other pathways that showed a copy number variation, it was “non-directional” or against the observed oncogenic activation [36].

This data implicates that copy number variation is a feasible method for identifying alterations in the PI3K/AKT/mTOR pathway in patients. The copy number variations observed in the PI3K/AKT/mTOR pathway also correlated with survival. Patients with two copies of wild-type PIK3CA *vs.* patients with a copy number gain or mutation in *PIK3CA* survived 59.3 months *versus* 28 months [36]. If a patient does not have any copy number variation or mutation in *PIK3CA*, *PIK3CB*, or *PIK3R4*, median survival was 80.4 months compared to patients with two or more alterations in different genes and thus a median survival of 18.2 months. The findings of this study indicate that not only can changes in the PI3K/AKT/mTOR pathway provide prognostic factors about survival, but also that genetic activation of the PI3K/AKT/mTOR pathway is an important characteristic of ovarian cancer.

Increasingly, clinicians and researchers believe that while genetic information is indicative of the genetic background of a patient’s tumor, functional readouts of alterations in a patient’s tumor can provide more information in directing future therapies. Identifying which patients are expected to respond to first-line chemotherapy treatment and future treatment can help guide therapy, that is, avoid therapies that will not benefit the patient. In a study that analyzed the ascites fluid of 88 patients with advanced ovarian cancer, the mutational status and phosphorylation status of members of the PI3K/AKT/mTOR pathway were analyzed. In patients that were chemotherapy naïve, there was a statistically significant increase in the level of phospho-p70S6K and p-AKT in the ascites fluid of patients who were classified as non-responders as compared to patients who had either a partial- or complete-response to first-line chemotherapy [73]. However, this increase in phospho-p70S6K and p-AKT did not correlate with corresponding mutations or amplifications in *PIK3CA*, mutations in *AKT2*, or loss of *PTEN.* While the study admits the lack of correlation could be the result of small sample size, it is also possible that there are other factors leading to over-activation of the p70S6K and AKT. Interestingly, in patients that did not respond to subsequent chemotherapy compared to patients who did, only phopsho-p70S6K was significantly elevated [73]. While there is no doubt that genetic alterations in *PIK3CA*, and *AKT*, or the loss of *PTEN* contribute to the role of the PI3K/AKT/mTOR pathway in the progression of ovarian cancer, this study suggests that that there are additional important factors that drive the PI3K pathway [73]. Furthermore, it is important to look at functional readouts of pathway activation, as elevated phospho-p70S6K levels may be indicative of chemoresistance.

Further studies are highly warranted in identifying the best scenarios for administering PI3K/AKT/mTOR pathway inhibitors. While PI3K/AKT/mTOR inhibitors are in trials for breast cancer and endometrial cancer, there have been limited clinical uses thus far in ovarian cancer. One of the first reports on clinical use of a PI3K/AKT/mTOR inhibitor in ovarian cancer looked at the utility of the dual-targeting strategy involving PI3K/AKT/mTOR and RAF/MEK/ERK pathways [74]. The PI3K/AKT/mTOR and RAF/MEK/ERK pathways are both heavily implicated in cancer progression. Also, both pathways have the ability to be activated by the RAS proteins and recent data has shown that when downstream AKT and mTOR are inhibited by pharmacologic agents, PI3K can activate mitogen-activation protein kinase (MAPK) via RAS [75]. There is concern that targeting one pathway will lead to quick resistance to that therapy, as the other pathway will compensate and take over-activation [76]. In this study of 236 patients, 32.2% received a combination of PI3K-pathway inhibitor and MAPK-pathway inhibitor, where 52.5% received just a PI3K-pathway inhibitor and 15.3% received a MAPK-pathway inhibitor [74]. In patients that had co-activation of both PI3K/AKT/mTOR and RAS/MEK/ERK that were treated with dual-inhibitors, all patients showed regression of the tumors varying between 2% and 64%. However, if the patients had a *PI3KCA* mutation with KRAS activation and only received an inhibitor to one of the two pathways, there was no response to therapy [74]. Considering the complexities of the PI3K/AKT/mTOR pathway, singleagent treatment is unlikely to be successful.

## 8. Conclusion

In the development and progression of ovarian cancer, it is clear that the PI3K/AKT/mTOR pathway plays an instrumental role. This role manifests itself in many unique ways presenting a complex picture for ovarian cancer. Alterations in the pathway can be the initializing event in aggressive high grade serous cancers or for low grade endometrioid type ovarian carcinoma. Mutations in the pathway can contribute to cellular proliferation, invasion and migration through modification of cell cycle inhibitors and MMPs. Additionally, changes in outside inputs, such as AMPK, sMEK, or FASN, can result in pathway changes without initiating genetic alterations in PI3K/AKT/mTOR family members. Multiple miRNAs can suppress or promote pathway activation in the same way as outside molecules.

Clinically, expression of p70S6K can help determine if a patient will respond to chemotherapy and copy number alterations can be prognostic for a patient’s survival. The PI3K/AKT/mTOR is a diverse pathway that affects equally diverse aspects of tumor development, progression, and patient survival. While targeting of PI3K/AKT/mTOR for treatment would seem to be an ideal strategy due to its importance, it will be difficult. Therapies will have to be given in combination and specifically tailored to each patient in order to have the most effect.
